# 

**Published:** 1913-01-15

**Authors:** 


### SUBJECT INDEX TO VOLUME LXVIII.

### EVENT AND COMMENT.

Another Taggart Patent Suit, 365.

Another Year, 570.

Chicago Dental Society, Annual Clinic, 106.

College Advertising, 1.

Crown and Bridge Work, a Curse to the Profession, 158.

Delayed by Floods, 157.

Dental Libraries, 54.

Dental Legislation, 210.

Dental Office Equipment, 418.

Dental Research Problems, 313.

Doctor of Dental Science Degree, 366.

Frank L. Platt, Resigns, 366.

Germicidal Tooth Powders, 469.

Important Dental Recognition, 262.

Index of Dental Literature, 365.

Institute of Dental Pedagogics, 105.

Medical Proffession Troubles, 3.

Pacific Gazette's New Editor, 418.

Perfecting the Gold Inlay, 263.

Prevention of Dental Caries, 314.

Proffession or Trade, 2.

Proffessional Versus, Commercial Ideals, 209.

Programme Suggestion, 520.

Progressive Clinics, 519

Proper Proximal Contacts, 157.

Pyorrhea and Its Treatment, 53.

Reorganization of the National Association, 54.

Reorganization of the New York State Society, 316.

Root Filling, 4.

Samuel A. Crocker, 261.

Taggart Boynton, Suit Decided, 108.

The Dental Relief Fund, 569.

The National Meeting, 417.

Troubles of an Examining Board, 571.

### FORMAL PAPERS.

Acute Alveolar Abscess, Its Pathology and Treatment, Rafael E. Terregrossa, 55.

A Frictional Tooth Polish, versus Gritty Tooth Powders and Pastes, J. P. Carmichael,212.

Analgesia in Dentistry, Wm. H. DeFord, 429.

A Sequel to Novocaine Injection, Simons Gooding, M. D., 131.

A Study of Saliva and its Action on Tooth Enamel, Joseph Head, 120.

A Successful Root Implantation, Vincenzo Guerini, 188.

Blood will prove a Man's Race, E. T. Reichert, 501.

Cause of Necrosis under Silicate Cements, 240.

Clasp and Bar Retention, S. F. Gilmore, 453.

Compressed Air for Desensitizing Dentine, W. T. Reeves, 437.

Control of Patients, Wm. C. Gillespie, 472.

Dental Economics, 406.

Dental Diseases and Our Responsibilities, N. S. Hoff, 265.

Dentition, B. B. O'Bannon, 394.

Effects of Stone Ground and Roller Flour on Decay of the Teeth and Nutrition, Thomas G. Read, 542.

Erotic Hallucinations due to Cocaine Pressure Anesthesia, 405.

Filling Root Canals with an Improved Paraffin Compound, Herman Prinz, 5.

Food, Health and Preventive Dentistry, Harvey W. Wiley, 220.

Food Vitamines, 549.

Formaldehyde, A Cure for Pyorrhea, G. A. Barnett, 456.

From the Outside Looking In, Jessie A. Jarvis, 551.

Institutional Dentistry; Methods; Results, Frederick A. Keyes, 25.

Look Out for the Pendulum, C. N. Johnson, 290.

Mastication, F. C. Husband, 29.

Many More Dentists Needed, Alfred P. Lee, 342.

Mouth Hygiene, W. G. Ebersole, 242.

Mushroom Poisoning, 229.

Nerve-Blocking as a Substiute for General Anesthesia in Surgical Operations, M. L. Harris, 584.

Obtunding Dentine by Local Applications, Elmore W. Elliot, 442.

Once More the Contact Point, C. N. Johnson, 173.

Oral Hygiene for Children, Wm. Lyon, M. D., 160.

Preliminary Note on Relief of Pain in Neuralgia, George O. Jarvis, M. D., 450.

Preservation of the Teeth, Norman Roberts, 347.

Preventive Dentistry, Henry A. Kelley, 317.

Pyorrhea Alveolaris: Its Pathology and Treatment, Thomas B. Hartzell, 61.

Pyorrhea Alveolaris, from the Standpoint of the General Practitioner, A. J. Cottrell, 375.

Pyorrhea Alveolaris, E. A. Lundy, 448.

Pyorrhea, Claude E. Hines, 385.

Red Rubber Stomatitis, J. W. Dent, L. D. S., 191.

Relations Between Carious Teeth and Malnutrition, Charles D. Carter, 496.

Removing Pulps by Arsenic Pastes and Cocaine under Pressure, J. W. Bryan, 572.

Restoration of Occlusion by the Casting Process, J. Lowe Young, 273.

Safeguarding Anesthesia, C. S. Tuttle, 338.

Some Facts—Chemical and Otherwise—About Dentifrices, Howard C. Kelly, 328.

Some Facts of Interest to Mouth Hygiene Workers, W. G. Ebersole, 288.

Some Forms of Ethical Quackery, E. O. Gillespie, 367.

Some Functions of the Dental Society, N. S. Hoff, 522.

Synthetic Cements, George C. Evans, 402.

The Contact Point and Interproximal Space, J. V. Conzett, 167.

The Cost of Dental Goods, W. J. Brady, 26.

The Dental Relief Fund, James McManus, 582.

The Distribution of the Nerves in the Pulp and Dentine, J. Howard Mummery, 192.

The Great Need, A. W. Thornton, 216.

The Influence the Dental Profession Sustains toward Civilization, L. J. McRea, 483.

The Neglect of the Gold Foil Filling, C. C. Johnson, 115.

The Painless Separation of Teeth, R. Ottolengui, 180.

The Philosophy of Mastication, George M. Niles, M. D., 162.

The Prophylactic Value of Typically Normal Occlusion, N. S. Hoff, 420.

Thoughts Relative to Artificial Dentures, I. B. Corolus, 234.

Tungsten and Its Dental Uses, L. E. Custer, 34.

Two Views of What the Dental Society Meeting Should Be, Two Editors, 590.

Where Do Our Patients Come in? H. F. Dickason, 38.

### BIBLIOGRAPHY.

American Text Book of Prosthetic Dentistry, 503.

Bayford's Handbook of Surgery, 457.

Biochemic Studies of Sulphocyanates, 83.

Broomell and Fischell's Anatomy of Mouth and Teeth, 133.

Dorlands Medical Dictionary, 505.

Exodontia, 504.

Ivy's Applied Anatomy and Oral Surgery, 292.

Practical Manual of Dental Casting, 352.

Prevention of Common Diseases in Childhood, 133.

Transactions, National Dental Association, 351.

OBITUARY.

Rudolph Beck, 246.

James R. Bell, 40.

Samuel A. Crocker, Sr., 293.

Elon G. Douglas, 246.

J. Austin Dunn, 246.

J. N. Farrar, 407.

Albert H. Fuller, 43.

Norman W. Kingsley, 198.

Wilbur F. Litch, 134.

Charles A. Meeker, 508.

Henry A. Smith, 506.

David C. Whalley, 84.

OBITUARY RESOLUTIONS.

Henry A. Smith, 594.
Thomas W. Clements, 595.

INDENTS.

A Call for Research Laboratories, 300.

Another Charge Against Formaldehyd, 600.

Acid Saliva, 49.

Advantage of the Porcelain Inlay 359.

A Method of Administering Nitrous Oxid and Ether, 295.

Antiquity of Bridge Work, 461.

Autogenous Vaccines in Chronic Joint Affections, 414.

A Perfect Denture, 416.

A Possible Danger in Amalgam Fillings, 203.

A View of Anesthesia, 560.

Bacterial Metabolism and Internal Medicine, 360.

Bridge Repaired Quickly Without Removing, 206.

Bromural in Dentistry, 206, 248,

Bulk in Porcelain Inlays, 357.

Burnishing Gold Inlays, 557.

Burnishing the Gold Inlay, 359.

Business Side of Dentistry, 354.

Care of Operator's Hands, 356.

Cement and Amalgam, 87, 355.

Clean Hands, 598.

Cleaning and Sterilizing Solution. 355.

Cleaning Old Vulcanite Dentures, 516.

Close Plated Platinum Utensils, 458.

Common Cold and Vaccine Therapy, 412.

Courteous Attitude, 91.

Cure Mouth Breathing, 460.

Cut Teeth from Throat, 44.

Death Under Nitrous Oxid and Oxygen, 254.

Defective Children in Cleveland Schools, 87.

Deforming Arthritis, 148.

Dental Diseases, A National Menace, 297.

Dental Education in United States as seen by a German Educator, 510.

Dental Recognition, 97.

Dentistry, Cause of Diseased Gums, 45.

Dentists and General Anesthesia, 145.

Dentistry, A Part of Public Health, 142.

Desensitizing Dentine, 358.

Digestibility of Bread, 145.

Digestibility and Food Value of Cheese, 95.

Disease Superstitions, 602.

Distilled Water, 597.

Drills, Reamers and Broaches, 250.

Effect of Heat on Metals, 93.

Effects of Treatment, 44.

Elongation of Posterior Teeth in Vulcanizing, 94.

Erosion of the Teeth, 415.

Etching on Steel, 250.

Extention for Prevention, 306.

Extirpating Pulps, 295.

Failures in Casting, 353.

Filling Children's Teeth, 202.

Formaldehyde Intoxication, 560.

Forsythe Dental Infirmary, 468.

Fusing Inlays on Platinized Gold, 140.

Gagging During Impression Taking, 47.

Get Busy, 151.

Gold Fillings in Artificial Teeth, 358.

Gold Shell Crowns, 353.

High Pressure Needle Packing, 295.

Home Made Appliances, 252.

How Root Canals Should Be Filled, 512.

Importance of Gum Integrity, 296.

Importance of Properly Mixing Cements, 465.

Impression Plaster, 557.

Increasing One's Efficiency, 605.

Infection from Dental Abscess, 297.

Ink for Writing on Glass, 601.

Inlays as Bridge Anchors, 564.

Inlay Cavity Preparation, 562.

Inlay Investment, 354.

Instructions for Brushing the Teeth, 200.

Intern Work Required for Medical Degree, 409.

Iodine in Mouth Washes, 144.

Is There Metabolism in Dentine, 465.

Justice for Jim Jam Jems, 458.

Life Conservation, 412.

Low Fusing Die Metal, 90.

Malposed Teeth as Bridge Supports, 462.

Matrices, 247.

Medical Research and Education, 410.

Mercuric Chloride in Surgery, 461.

Method of Reinforcing Plaster, 136, 304.

Missing Link, 515.

Mortality of Dentists Compared, 141.

Moving Mature Teeth, 463.

National Dental Association, 311.

New Method of Cauterizing with Silver Nitrate, 87.

Novocaine, 149.

Novrenin, 565.

Observations by Brother Bill, 91.

Occupation of Parents and Child Birth, 148.

Oral Hygiene in Cincinnati Schools, 153.

Oral Hygiene Teachers at Loggerheads, 604.

Our Work, 100.

Oxygen Poisoning, 144.

Peculiar Case, 90.

Phylacogens Again, 145.

Placing Chloro-Percha, 557.

Poisoning by Analine, 557.

Practical Suggestions, 137.

Pressure Anesthesia, 559.

Prevention, 90.

Preparing Wax Pattern for Investment, 357.

Prevent Silver Oxidation, 601.

Preventing Splint Rivets, Showing 92.

Prevention of Cancer, 92.

Professional Advice, 415.

Prophylaxis of Cancer, 147.

Professions Overcrowded, 136.

Property versus Life, 459.

Purified Drinking-Water, 602.

Quinine and Urea Hydrochlorid, 559.

Red Rubber Poisoning, 93.

Removing Deposits from German Silver, 255.

Removing Enamel for Crowns, 305.

Removing Plaster from Vulcanite Plate, 510.

Rosin Solution as a Root Filling, 137.

Rubber Cements, 416.

Saddle Bridges, 411.

Smoking in Winter Injurious, 48.

Simple Means of Polishing Fillings, 253.

Simple Method of Polishing Instruments, 510.

Simple Method of Sterilizing Rubber Gloves, 596.

Solder Bench, 251.

Status of Infirmary Work in Chicago, 201.

Sterilization of Drinking-Water on Small Scale, 596.

Sterilizing Rubber Gloves, 596.

Taking the Bite, 356.

Technic of Accurate Inlay Matrix, 253.

Teething as A Cause of Disease, 513.

The Contact Point, 565.

The Gingival Wall, 305.

The Gold Band in Crown Work, 200.

The Specialists, 301, 414.

To Clean Old Dentures, Etc., 601.

To Make Ideal Gold Dummies, 249.

Tragacanth, Jelly, 558.

Treating Pyorrhea, 602.

Turkish Baths, 45.

Unclean Mouths A Menace, 303.

Undoing the Dentist' 603.

Vacuum versus Compressed Air, 89.

Value of a Complete Diagnosis, 250.

Varnish for Plaster Casts, 354.

Vinegar for Softening Plaster, 358.

Vitiated Saliva, 48.

Ward's Low Fusing Metal, 90.

Wax Inlay Pattern, 50.

Whole Meal Bread, 138.

Why A National Association, 88.

Wisdom Teeth at Fault, 47.

AUTOGRAPHIC.

Allen, C. C., 153.

Allen, C. E., 249.

Ames, W. V.-B., 465.

Barker, D. N., 557.

Barnett, G. A., 456.

Beach, John, 576.

Beck, Rudolph, 246.

Bell, James R., 240.

Best, E. S., 357.

Black, A. D., 305, 307.

Bowman, G. A., 510.

Brady, W. J., 26.

Brown, C. E., 358.

Bryan, J. W., 572, 580.

Bryant, Lester, 565.

Buckley, J. P., 297.

Buxbaum, Dr., 208.

Callahan, J. R., 137.

Carmichael, J. P., 212.

Carpenter, E. R., 463.

Carter, C. D., 496.

Case, C. E., 464.

Cattell, D. M., 579.

Clements, Thos. W., 595.

Clottky, H., 93.

Conzett, J. V., 167.

Cook, G. W., 566.

Coolidge, E. D., 51.

Corolus, I. B., 234.

Cottrell, A. J., 375.

Craft, W. H., 295.

Crocker, Samuel, A., 293.

Crossland, J. H., 359, 557.

Crouse, J. N., 49.

Cruise, R. J., 201.

Custer, L. E., 34.

Dalbey, W. C., 89.

Darter, E. T., 303.

DeFord, W. H., 93.

Dent, J. W., 93, 191.

Dickason, H. F., 38.

Douglas, E. G., 246.

Dunn, J. A., 246.

Ebersole, W. G., 242, 288, 311, 442.

Endelman, Julio, 462.

Evans, George, C., 402.

Evans, W. A., 90.

Farrar, J. N., 407.

Fellman, W. O., 87.

Flood, A. M., 89.

Fuller, A. H., 43.

Gaylord, E. S., 254.

Gethro, F. W., 564.

Gillespie, E. O., 367.

Gillespie, W. C., 472.

Gilmore, F. S., 453.

Gooding, S., M.D., 131.

Grieves, C. J., 602.

Guerini, Vincenzi, 188.

Harris, M. L., 584.

Haskell, L. P., 47.

Hartzell, T. B., 61, 297, 534.

Head, Joseph, 120.

Hermann, Chas., 515.

Hetrick, F. O., 311.

Hines, Claude E., 385.

Hinkins, J. E., 51.

Hoff, N. S., 265, 420, 522.

Holder, H. A., 578.

Horsely, J. W., 138.

Hunt, G. E., 304.

Husband, F. C., 29.

Jarvis, G. O., M.D., 450.

Jarvis, J. A., 551.

Jenkins, N. S., 358, 460.

Johnson, C. C., 115.

Johnson, C. N., 173, 290, 300, 301.

Johnson, Eugene, 579.

Junkerman, G. S., 205.

Kahn, Max, 83.

Kelley, H. A., 317.

Kelly, H. C., 328.

Keyes, F. A., 25.

Kingsley, N. W., 198.

Kirk, E. C., 144, 150.

Kohn, K., 512.

Lederer, W. J., 356.

Lee, A. P., 248, 342.

Leonard, N. C., 574.

Lewis, H. R., 354.

Libbey, J. A., 557.

Litch, Wilbur, F., 100, 134.

Little, J. F., 93.

Lowenthal, Wm., 206.

Lundy, E. A., 448.

Lyon, Wm., M.D., 160.

Mares, H. A., 305.

McManus, J., 582.

McRea, L. J., 483.

Meeker, Charles A., 508.

Mertens, Alex, 467.

Miller, D. W. N., 559.

Moon, W. D. N., 250.

Mueller, A., 512.

Mummery, J. Howard, 192.

Neil, Ewell, 577.

Niles, Geo. M., M.D., 162.

Noel, L. G., 577.

North, Gustav, 137.

O'Bannon, B. B., 394.

Ottolengui, R., 180, 203.

Olow, John, 255.

Palmer, C., 251.

Pedley, J. K., 295.

Prime, J. M., 137, 305.

Price, W. A., 141.

Prinz., H., 5.

Puterbaugh, P. G., 354.

Read, Thos. G., 542

Reeves, W. T., 437.

Reichert, E. T., 501.

Roach, F. E., 412.

Roberts, C. M., 92.

Roberts, Norman, 347.

Ryan, Frank J., 87.

Sabin, C. R., 416.

Sanders, M. E., 601.

Schwartz, F., 253.

Skinner, F. H., 200.

Smith, Ray E., 597.

Smith, H. A., 506, 594.

Spence, Stewart J., 601.

Talbott, E. S., 45.

Taylor, C. H., 580.

Taylor, L. C., 336.

Terregrossa, R., 35.

Thornton, A. W., 216.

Tracy, Wm. D., 414, 415.

Trueman, W. H., 354, 355, 357, 605.

Tuttle, C. S., 338.

Van Woert, C. T., 558.

Wallac, J. Sim., 133.

Watkins, S. G., 47.

Whalley, D. C., 84.

Wiley, Harvey W., 220.

Williger, Prof., 249.

Wood, R. J., 353.

Young, J. Lowe., 273.

### ANNOUNCEMENTS.

American Society of Orthodontists, 257.

California State Society, 257.

Chicago Dental Society Officials, 101.

Colorado State Society, 257.

Connecticut Examining Board, 517.

Dental Motion Picture, 103.

Dental Meetings in April, 156.

Dental Meetings for May, 207.

Dental Relief Fund, 610.

Examinations for Army and Navy, 467.

First Pan-American Congress, 362.

Fourth International Hygiene Congress, 308, 310.

Georgia State Meeting, 257.

Idaho State Board, 307.

Illinois State Board, 258, 518.

Indiana State Board, 258, 517.

Institute of Pedagogics, 568.

Institute of Pedagogics Officers, 156.

Maryland State Board, 517.

Massachusetts State Board, 517.

Michigan State Board, 258, 517.

Minnesota State Meeting, 257.

Mississippi State Meeting, 258.

Montana State Board, 307.

National Association Annual Meeting, 208, 258, 307.

National Association Examiners, 307.

National Dental Association, 566.

National Falculties Association, 307.

National Hygiene Association, 310.

National Dental Relief Association, 51.

New Jersey Examiners, 568.

New Jersey State Meeting, 307.

Northern Michigan Society, 258.

Northern Indiana Society, 256, 468.

Northern Ohio Association, 208, 256, 258.

Ohio Examining Board, 258.

Ohio State Dental Society, 567.

Ohio State Society, 255, 467.

Panama Pacific Congress, 606.

Pan-Pacific Congress, 101, 363, 518.

Pennsylvania Examiners, 258.

Proceedings National Association, 567.

Sixth International Congress, 468.

Sixth Internatioal Dental Congress, 607.

Southern Branch National Association, 207, 307.

Tennessee State Meeting, 258.

Texas State Association, 156.

The Panama Pacific Dental Congress, 606.

Washington University, 207.

Western Dental Journal, 208.

West Virginia Examiners, 258.

West Virginia State Society, 256, 308.

Wisconsin Examiners, 259, 308.

### ANNOUNCEMENTS.

National Dental Relief Fund. Dear Doctor: Are you acquainted with the effort of the National Dental Association to establish a fund for the relief of its aged and unfortunate members?

Are you in full sympathy with that movement?

If familiar with the scheme thus far, you are aware that the first step was the endeavor during the present year to carry out the plan endorsed by the National Dental Association at Cleveland in 1911. That plan was to ask each State society to increase its annual dues one dollar per member, and set aside the sum thus obtained for the Relief Fund The committee appointed at Cleveland endeavored to get the question before all the State societies at their last annual meetings, but coming at the same time that we were asking them to increase their dues for the purpose of entering the National Dental Association under the reorganization, we met with much opposition.

The question was not put to a vote in a number of cases, because it was feared we would hinder the reorganization movement. We were received with words of encouragement everywhere, but thus far we have only two States committed to our plan, namely: Tennessee and Colorado. Several of the other States are almost presuaded, and we hope they will vote the measure at their next meetings; therefore, we shall keep up our efforts to carry out the original plan. We have been assured that this plan will not carry in some States for various and sundry reasons, and many opposed to it have asked to be allowed to make voluntary annual contributions instead. Some few noble spirits have already set the example by sending on to the committee their first assessment. This encourages us to come to you, and we ask you to join this generous few and agree to give annually a small sum to the Relief Fund. This is not to be something "thrown away, never-to-be-heard-of-again," but it is going to create a fund to insure our members against want in sickness and old age. You may yourself become a beneficiary, or may live to see it help some dear professional friend in time of distress.

Will you, then, send us your name and the sum you are willing to contribute, or will you not save time and correspondence by enclosing your check at once for the first annual payment?

Make your check payable to H. B. McFadden, of Philadelphia, Treasurer of the National Dental Association. You may rest assured that the distribution of the Relief Fund will be in the hands of those who will thoroughly investigate all applications for help. Every precaution will be taken to guard the funds and utilize them for no other purpose than the relief of worthy dentists.

Will you not ask your professional friends to join you in this movement? Will you not lend us your aid and influence to get our original plan adopted by your state society? Will you not call upon the members of the profession to contribute what they can? Will you not advise us, giving names of those you call upon who feel they cannot contribute, saving us further correspondence and them future annoyance?

Fraternally yours,
L. G. NOEL,
E. S. GAYLORD,
W. T. CHAMBERS,
National Relief Committee.

